# Ruptured Internal Iliac Artery Aneurysm Presenting as Syncope and Left-Sided Abdominal Pain: A Rare Cause of Collapse in the Emergency Department

**DOI:** 10.7759/cureus.94338

**Published:** 2025-10-11

**Authors:** Ritesh Yadav, Dhananjay Singh, Nabila Natasya, Hussain Ghadir

**Affiliations:** 1 Emergency Department, South Tyneside and Sunderland National Health Service (NHS) Foundation Trust, South Shields, GBR

**Keywords:** abdominal pain, aneurysm rupture, collapse, emergency medicine, endovascular embolization, hypotension, internal iliac artery aneurysm, retroperitoneal hemorrhage, syncope, vascular emergency

## Abstract

Ruptured internal iliac artery aneurysm (IIAA) is a rare but life-threatening vascular emergency that can mimic gastrointestinal or urological conditions. A 61-year-old man presented to the emergency department with sudden, severe left iliac fossa and groin pain followed by two episodes of collapse. On arrival, he was hypotensive (88/68 mmHg) and pale, with left-sided abdominal tenderness but no pulsatile mass. The bedside ultrasound was unremarkable, but his condition deteriorated despite fluid resuscitation. Laboratory tests showed low hemoglobin and raised lactate. Contrast-enhanced computed tomography (CT) revealed a ruptured left IIAA measuring 7 × 6.7 cm with extensive retroperitoneal hemorrhage. The patient underwent urgent endovascular embolization and stent placement, with successful aneurysm exclusion and recovery. This case emphasizes the need to consider vascular causes in patients presenting with collapse and lower abdominal pain, even when initial imaging is inconclusive. Early CT imaging and prompt endovascular management are crucial for survival in such rare presentations.

## Introduction

Isolated internal iliac artery aneurysm (IIAA) is a very rare diagnosis with a non-specific presentation that could be missed in the emergency department. It is reported that the incidence of IIAAs is approximately only 0.04%-7% of all aortoiliac aneurysms [1,2]. A study conducted in China reported that IIAAs were found in 1.2% of patients with acute aortic dissection who presented to the emergency department [3]. Rupture of IIAAs could manifest in several symptoms and often mimics other acute conditions, such as pain in the left leg and hip region, shock, abdominal pain, prostate necrosis, obstructive micturition, back pain, and neurological deficits in the leg [1,4]. Some cases also report asymptomatic cases with only hypotension and reduced kidney function on laboratory findings [4].

IIAAs can be easily missed due to their variable clinical presentations and limitations of bedside imaging. Although point-of-care ultrasound (POCUS) demonstrates high sensitivity (99%) and specificity (98%) [5] for detecting abdominal aortic aneurysms (AAAs) and remains a key bedside diagnostic tool, its accuracy can be reduced by overlying bowel gas or the deep anatomical location of the internal iliac artery, making visualization challenging [6,7]. A contrast-enhanced computed tomography (CT) scan is often required for definitive diagnosis and to help plan the treatment in this case. A new guideline from the European Society for Vascular Surgery (ESVS) in 2024 recommended performing a CT scan, especially in the suspicion of rupture of IIAAs [7]. Clinical guidelines recommend that emergency surgical repair be performed within 90 minutes of patient arrival to optimize outcomes. Timely diagnosis in the emergency department, which is expected to be done in less than 30 minutes, is critical to achieving a favorable prognosis in such cases [7,8].

We present a rare case of a ruptured IIAA in a 61-year-old male patient who presented with syncope and left iliac fossa pain, initially suspected to be gastrointestinal in origin. This case underscores the importance of maintaining a high index of suspicion for vascular causes in patients presenting with collapse and abdominal pain, especially when initial investigations are inconclusive.

## Case presentation

A 61-year-old man presented to the South Tyneside District Hospital Accident and Emergency (A&E) department with a complaint of collapse and left-sided abdominal pain. The abdominal pain was sudden in nature, described as a sharp, severe pain on the left side of the groin, along with the urge to go to the toilet. Due to the severity of the pain, the patient had a syncopal episode on the toilet and was found by his friend lying on the floor, unresponsive and pale. When helped to get up, the patient collapsed for the second time. The patient arrived in the emergency department fully conscious and alert. The patient could describe the event and explained that he felt dizzy when he was trying to stand. Pain was located around the left iliac fossa and radiated to the groin. Witnesses denied any seizure activity during the collapse. The patient denied any urinary symptoms and denied any change in bowel habit. The patient also denied any chest pain or abdominal distention. No bleeding is noted anywhere. The patient had a history of hypertension and right-sided cerebrovascular accident and was housebound due to agoraphobia. His routine medication includes atorvastatin, ramipril, clopidogrel, bisoprolol, and amlodipine. The patient reported consuming approximately 22 units of alcohol per week and had been a smoker for 48 years.

During the physical examination, it was found that his blood pressure was 88/68 with a heart rate (HR) of 85 bpm, oxygen saturation of 98%, and temperature of 35.5°C. Tenderness on the left side of the abdomen was found during examination, with no sign of guarding and no palpable mass. A POCUS scan was performed bedside in A&E to rule out AAA and abdominal free fluid; however, the abdominal aorta measured within normal limits (≤3 cm in diameter), and no free fluid was visualized. The patient was not improving despite fluid resuscitation; instead, blood pressure dropped to 65/45. Blood tests demonstrated evidence of acute blood loss and tissue hypoperfusion, including a drop in hemoglobin, mild microcytosis, thrombocytopenia, and elevated lactate, with mild hyponatremia. Other parameters are within normal limits (Table 1). ECG showed tachycardia with a rate of 186 bpm.

**Table 1:** Laboratory result L: low; H: high; WBC: white blood cells; Hb: hemoglobin; Hct: hematocrit; MCV: mean corpuscular volume; MCH: mean corpuscular hemoglobin; PLT: platelets; pH: acidity/alkalinity; pO₂: partial pressure of oxygen; pCO₂: partial pressure of carbon dioxide; HCO₃⁻: bicarbonate; BE: base excess; eGFR: estimated glomerular filtration rate; GGT: gamma-glutamyl transferase; ALT: alanine transaminase; ALP: alkaline phosphatase; CRP: C-reactive protein; PT: prothrombin time; PTT: partial thromboplastin time

		Result	Normal range
Full blood count	WBC	10.88 x 10^9^/L	3.6-11.0 x 10^9^/L
	Hb	114 g/L (L)	130-180 g/L
	Hct	0.335 L/L (L)	0.40-0.54 L/L
	MCV	108.1 fL	80-100 fL
	MCH	36.8 pg	27-32 pg
	PLT	121 L x 10^9^/L	140-400 x 10^9^/L
Coagulation	PT	13.9 sec	9-13 sec
	PTT	30.2 sec	22-36 sec
	Fibrinogen	2.29 g/L	1.50-4.50 g/L
Venous blood gas	pH	7.27 (L)	7.32-7.43
	pO_2_	2.4 kPa (L)	3.5-5.5 kPa
	pCO_2_	5.3 kPa	4.5-6.0 kPa
	HCO_3_^-^	18.1 mmol/L	22-28 mmol/L
	BE	-8.9 mmol/L	-2 to +2 mmol/L
	Lactate	6.2 mmol/L (H)	0.5-2.2 mmol/L
	Glucose	6.3 mmol/L	3.5-7.8 mmol/L
Chemistry	Sodium	128 mmol/L (L)	135-145 mmol/L
	Potassium	3.8 mmol/L	3.5-5.0 mmol/L
	Bicarb	15 mmol/L (L)	22-28 mmol/L
	Urea	2.0 mmol/L (L)	2.5-7.8 mmol/L
	Creatinine	75 mmol/L	60-110 µmol/L
	eGFR	>90 mL/min/1.73 m²	>90 mL/min/1.73 m²
	Calcium	1.77 mmol/L (L)	2.20-2.60 mmol/L
	Magnesium	0.80 mmol/L	0.7-1.0 mmol/L
	Phosphate	1.38 mmol/L	0.8-1.5 mmol/L
	Total bilirubin	3 µmol/L	3-21 µmol/L
	GGT	192 U/L	<55 U/L
	ALT	12 U/L	<45 U/L
	ALP	74 U/L	30-130 U/L
	CRP	0.7 mg/L	<5 mg/L

**Figure 1:**
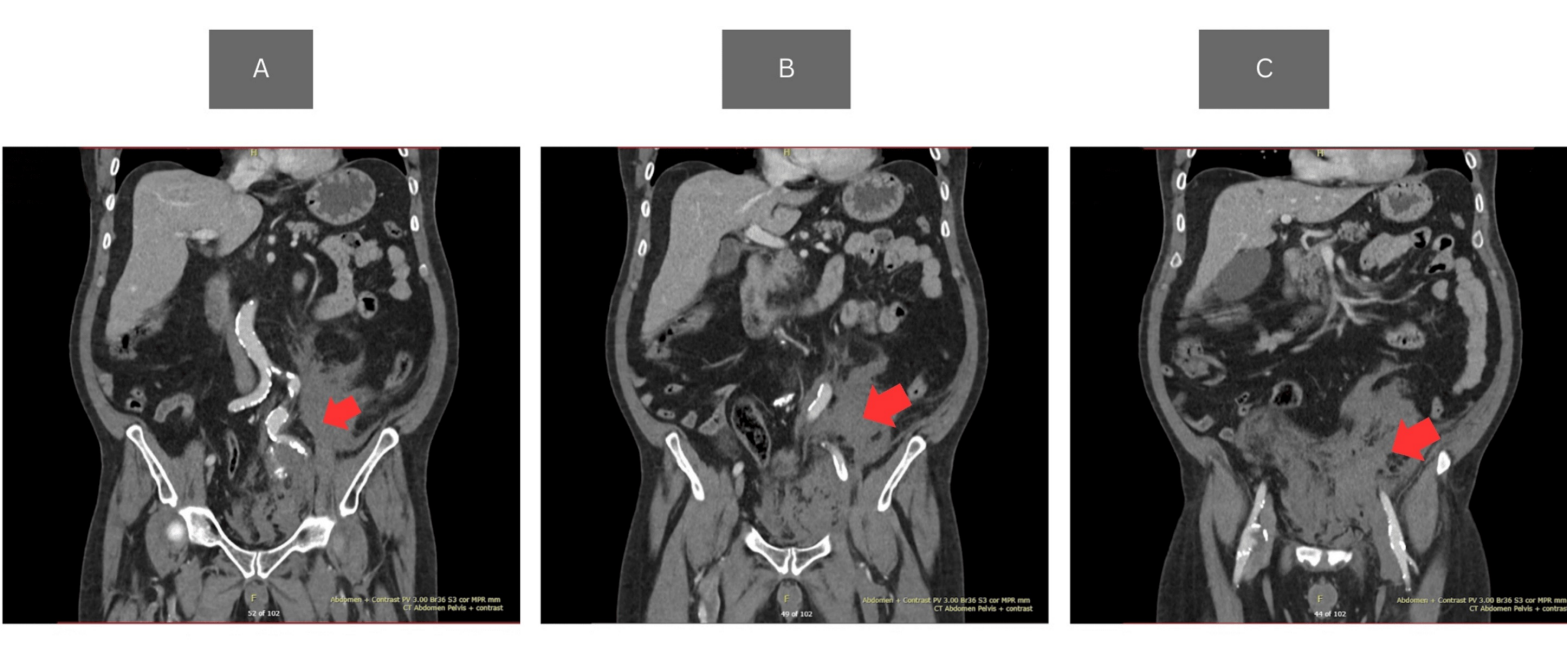
CT abdomen with contrast enhanced (A) Abrupt interruption of the vessel (red arrow); (B) progressive collapse and irregularity of ruptured artery segment (red arrow); (C) absence of distal internal iliac artery flow beyond the rupture site (red arrow) CT: computed tomography

Suspicion of internal bleeding was raised based on the physical examination and blood results. A contrast-enhanced CT scan of the abdomen and pelvis was performed approximately 45 minutes after arrival, including arterial and portal venous phases, to evaluate for intra-abdominal bleeding and vascular abnormalities. It was found on the CT scan that there was a large ruptured left internal iliac aneurysm. The size of the aneurysm was up to 7 x 6.7 cm, and there was a layering of blood products with different densities along with marked surrounding inflammatory fat stranding and hyperdense fluid surrounding the aneurysm tracking into the left retroperitoneal superiorly and inferiorly into the pelvis. There was a large hematoma in the suprapubic region and pelvis anterior to the aneurysm. Aortoiliac atherosclerotic calcifications are noted, and there was a 3 cm aneurysm in the right femoral artery (Figure 1).

**Figure 2:**
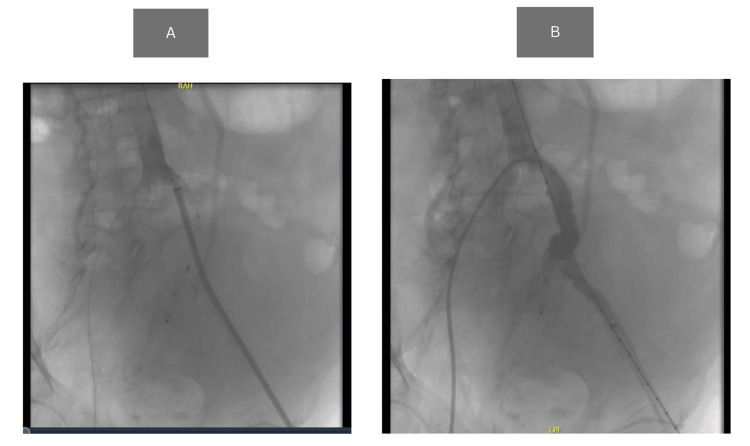
Angiography (A) Initial angiogram showing the site of internal iliac artery rupture with contrast extravasation and vessel irregularity; (B) post-intervention angiogram revealing successful deployment of an endovascular stent graft

As bleeding was found, fluid resuscitation was stopped to achieve a relative hypotension effect. A vascular surgery consultation was done, and the patient was sent to the Sunderland Royal Hospital for further management. Emergency embolization of the left internal iliac artery and stent placement were done under the vascular surgery department. The patient was put under local anesthesia with the USS-guided entry of the left common femoral artery (CFA). Amplatzer vascular plug II (20 mm) was deployed at the origin of the left internal iliac artery to occlude blood flow into the aneurysm. A Gore Viabahn covered stent graft (12 × 200 mm limb) was deployed in the common and external iliac arteries to exclude the origin of the internal iliac artery and maintain distal perfusion. The final angiographic run demonstrated a good seal of the artery with no filling of the aneurysm sac (Figure 2). Pedal pulse is palpable at the end of the procedure. The patient was kept in the ICU for two days after the procedure and moved to the ward for hospitalization for two days before finally being discharged.

## Discussion

This case describes a 61-year-old male patient presenting with collapse and acute left-sided abdominal pain that radiates to the groin without any urinary symptoms, who was then ultimately diagnosed with a ruptured left IIAA. IIAAs are mostly asymptomatic before rupture. Symptoms are usually present when there is pressure on the surrounding area, such as the ureter, iliac vein, obturator nerve, colon, and small bowel [9-11]. Previous systematic reviews that studied 70 case reports of IIAAs from 1947 to 2004 show that most patients presented with abdominal pain, which accounted for 31.7% followed by urinary symptoms, lumbar-sacral pain, and groin pain [10]. Other symptoms indicating rupture of IIAAs include hemodynamic collapse, pallor, back pain, abdominal pain, and distension [7]. A recent review using nine reported cases from 1991 to 2023 shows that 44.4% cases showed abdominal pain as a symptom, followed by neurovascular problems in the leg and accidental findings during laboratory and imaging tests, which accounted for 22.2% each, and one case presented with back pain and micturition problems [3]. Both reviews are consistent with reporting abdominal pain as the most common presentation, which is similar to our patient's presentation.

Risk factors for IIAAs are similar to AAA [2-8]. Our patient has several risk factors that possibly lead to IIAA's diagnosis. A study shows that 94% of patients who are diagnosed with IIAAs are male at an average age of 68 years old. Most of them have no symptoms before it ruptures [2]. This risk factor matches our patient characteristics. Additionally, being a smoker for 48 years significantly increases the risk of developing IIAAs. It is well established that smoking is the strongest risk factor for AAA, with a risk tripled in men of White ethnicity [7]. The patient also has hypertension, which contributes to developing IIAAs [7,8]. The history of non-hemorrhagic stroke in our patient also represents atherosclerotic disease, which is also considered a strong risk factor for vascular aneurysm [7,8]. This is also later evidenced by the finding of calcifications in the aortoiliac vessels and a concurrent 3 cm right femoral aneurysm. Other recorded risk factors for IIAAs include infection, trauma, connective tissue disorder, fibromuscular disease, Kawasaki disease, and Behcet's disease [9].

A notable aspect of this patient is his agoraphobia and housebound status, which likely contributed to delayed recognition and presentation. Being largely confined to his home may have prevented timely medical review for underlying vascular disease, including his known hypertension and atherosclerotic risk factors. Patients with agoraphobia may also underreport or tolerate progressive symptoms, presenting only when complications become acute and potentially life-threatening. This highlights the importance of considering social and psychological factors when evaluating patients at high risk. Given his vascular risk profile, current guidelines recommend screening with ultrasound (US) for high-risk individuals, with follow-up imaging of iliac artery aneurysms every three years for aneurysms measuring 2.0-2.9 cm and annually for aneurysms 3.0-3.4 cm if proven to have IIAAs [2,7]. This case underscores the need for comprehensive vascular assessment and surveillance, particularly in patients who are socially isolated or have mental health conditions that may limit access to healthcare.

According to the guideline management of abdominal aortoiliac aneurysm by ESVS, US is recommended as the first-line modality in diagnosing small AAA including IIAAs, with high sensitivity and specificity [7]. They recommend measuring outer-to-outer (OTO) in using the anteroposterior plane with consistent caliper placement to measure the diameter of the diseased aorta [7]. One of the case reports showing the US findings for ruptured IIAAs includes a fusiform cystic mass adjacent to the right internal iliac artery in the right pelvic cavity with an anechoic cystic portion that showed swirling turbulent flow on color Doppler US, which is showing the aneurysm [12]. Another report shows free fluid in the intra-abdominal cavity along with a large aneurysm in the right lower abdomen, in the case of ruptured IIAAs [13]. In this case, despite being performed by a highly experienced and POCUS-certified doctor, our examination did not detect the aneurysm. POCUS/Focused Assessment with Sonography in Trauma (FAST) is primarily designed to assess free fluid in the peritoneal cavity, and for minimal intraperitoneal bleeding (>200 mL), it has a reported sensitivity of up to 85% [14]. However, the internal iliac artery is located in the retroperitoneal space, and bleeding from this artery is unlikely to present in the peritoneum [6,7]. Therefore, despite the negative POCUS finding, a contrast-enhanced CT scan of the abdomen and pelvis was required to confirm the diagnosis. Guidelines recommend CT angiography for both diagnosis and treatment planning of ruptured IIAAs [7].

Initial management of ruptured IIAAs plays an important role in better patient outcomes. Both National Institute for Health and Care Excellence (NICE) and ESVS guidelines recommended that the patient should be allowed permissive hypotension [7,15] to allow clotting formation on the vascular wall and avoid the development of iatrogenic coagulopathy [7,14,16]. It is predicted that each liter of fluid volume added for resuscitation before aortic clamping could increase the relative risk of perioperative mortality by 60% [15]. It is recommended that the maximum pre-hospital fluid volume given to the patient is 500 mL with a target systolic blood pressure (SBP) around 70-90 mmHg [7]. It is also recommended to do resuscitation using red blood cells and fresh frozen plasma with a ratio of 1:1 [7].

Definitive management for rupture of IIAAs includes open vascular repair and an endovascular approach. Recent guidelines recommend performing endovascular repair as the first choice in patients with suitable anatomy [7]. Our patient underwent endovascular repair with stent placement on the same day of diagnosis. After surgery, it is important to monitor intra-abdominal pressure (IAP) for early diagnosis of abdominal compartment syndrome. In a patient with IAP measured >30 mmHg or >20 mmHg with organ failure, urgent abdominal decompression needs to be done [7].

## Conclusions

Early recognition of ruptured IIAAs and a correct resuscitation approach in the emergency department is crucial for prompt intervention and improved patient outcomes. In patients presenting with hemodynamic instability and non-specific truncal pain, ruptured IIAAs should be considered as a differential diagnosis to ensure timely diagnosis and life-saving management.
